# Glycosylation Modulates the Structure and Functions of Collagen: A Review

**DOI:** 10.3390/molecules29071417

**Published:** 2024-03-22

**Authors:** Igor Tvaroška

**Affiliations:** Institute of Chemistry, Slovak Academy of Sciences, 845 38 Bratislava, Slovakia; chemitsa@savba.sk

**Keywords:** collagen, structure, collagen hydroxylases, collagen glycosyltransferases, catalytic mechanism, glycosylation functions, collagen glycosylation-related diseases

## Abstract

Collagens are fundamental constituents of the extracellular matrix and are the most abundant proteins in mammals. Collagens belong to the family of fibrous or fiber-forming proteins that self-assemble into fibrils that define their mechanical properties and biological functions. Up to now, 28 members of the collagen superfamily have been recognized. Collagen biosynthesis occurs in the endoplasmic reticulum, where specific post-translational modification—glycosylation—is also carried out. The glycosylation of collagens is very specific and adds β-d-galactopyranose and β-d-Glc*p*-(1→2)-d-Gal*p* disaccharide through β-*O*-linkage to hydroxylysine. Several glycosyltransferases, namely COLGALT1, COLGALT2, LH3, and PGGHG glucosidase, were associated the with glycosylation of collagens, and recently, the crystal structure of LH3 has been solved. Although not fully understood, it is clear that the glycosylation of collagens influences collagen secretion and the alignment of collagen fibrils. A growing body of evidence also associates the glycosylation of collagen with its functions and various human diseases. Recent progress in understanding collagen glycosylation allows for the exploitation of its therapeutic potential and the discovery of new agents. This review will discuss the relevant contributions to understanding the glycosylation of collagens. Then, glycosyltransferases involved in collagen glycosylation, their structure, and catalytic mechanism will be surveyed. Furthermore, the involvement of glycosylation in collagen functions and collagen glycosylation-related diseases will be discussed.

## 1. Introduction

The extracellular matrix (ECM) is a non-cellular component of the body existing within all tissues and organs and is responsible for their maintenance and integrity. ECM consists of diverse macromolecules. Proteins or glycoproteins, such as collagens, elastin, laminins and tenascins, proteoglycans, glycans, and polysaccharides form a dynamic 3-dimensional network providing structural support for the cells and tissues [1,2]. These macromolecules modulate many cellular processes, such as cell signaling functions, properties, and phenotype. ECM coordinates interactions between cells in organs and tissues using inside–out and outside–in signals [3]. Interactions of ECM components with their cell receptors play significant roles in health and diseases [4,5,6].

Collagens, with over 30 % of the total proteins, are the most dominant protein in the human body and comprise 80–90 % of ECM as the principal constituent [6,7,8]. The characteristic structural feature of collagens is the presence of a triple helix consisting of repeated amino acid triplets (XaaYaaGly)_n_. In vertebrates, the collagen family comprises 28 diverse members [9,10,11] divided into several subfamilies based on their supramolecular architecture and function. Collagens are present in soft and hard connective tissues such as the skin, tendons, cartilage, bone, teeth, and corneas [1]. They provide the required tissue integrity and are involved in the other cell processes. Defects in collagens structures, either autoimmune or genetic origin, are associated with most 40 collagen diseases [12,13] of various severity, such as bone and cartilage abnormalities, skin alternation, visual defects, hearing loss, vessel abnormalities, kidney diseases, lung diseases, and cancer [14,15,16,17,18].

Before forming a triple helix, a collagen single chain undergoes post-translational modifications (PTMs) in the endoplasmatic reticulum (ER). The PTM starts with hydroxylation of the proline and lysine residues, which thermally stabilizes the triple helix [19]. Then, some of the formed hydroxylysine (Hyl) residues are glycosylated. The unique glycosylation of collagen involves linking β-d-galactopyranose via β-linkage to Hyl and adding glucopyranose to the C2 position of galactopyranose by α-linkage. The resulting *O*-glycan on collagen is the simple β-d-galactopyranose or α-d-glucopyranosyl-(1→2)-β-d-galactopyranose disaccharide [20]. Although collagen has been the subject of numerous studies for about 100 years, progress in understanding the glycosylation of collagens commenced in the last two decades, and many aspects remain unclear [21]. Moreover, collagen glycosylation was recently associated with autoimmune diseases like rheumatoid arthritis [22], systemic sclerosis [23], and cancer [24]. Recently, various crystal structures of human collagen lysyl hydroxylase 3 (LH3, decoded by *PLOD3* gene) that are responsible for the transfer of glucopyranose to the galactosylated Hyl have been solved [25,26,27,28].

Several reviews and books have been published on various collagen structures, biosynthesis, biological functions, and application aspects [10,11,12,29,30,31,32,33,34,35,36,37]. Therefore, this is not intended to be an exhaustive review of all collagen studies, which are only briefly discussed. Instead, the review will focus on collagens’ less understood aspects, namely glycosylation. The review starts with a short overview of collagen structure and biosynthesis. Then, current knowledge about collagen glycosylation, enzymes involved in glycosylation, and their catalytic mechanisms will be discussed. Finally, the question of how alternation in glycosylation is associated with structural changes and various biological and pathological processes will be addressed.

## 2. Collagen Structure

Collagens interact with various adhesion molecules associated with multiple biological processes. A systematic understanding of the structure of collagens is crucial for understanding their biological functions. In the last 40 years, the knowledge about collagen’s structure has considerably progressed and has been described in several reviews [7,9,10,11,29,30,31,33,38,39,40,41,42]. The characteristic feature of collagens is triple helix formed by three parallel polypeptide chains.

In vertebrates, the collagen superfamily consists of 28 members with numbers denoted by Roman numerals [9,10,43]. Collagens are classified based on their domain structure and supramolecular organization. They can be grouped into fibril-forming collagens (types I, II, III, V, XI, XXIV, and XXVII); FACIT—fibril-associated collagens (types IX, XII, XIV, and XX); network-forming collagens (types IV, VI, VII, VIII, and X); FACIT-like collagens (types XVI, XIX, XXI, and XXII); transmembrane collagens (types XIII, XVII, XXIII, and XXV); multiplexin collagens (types XV and XVIII); and other molecules with collagenous domains (types XXVI and XXVIII) [43]. Collagens can be homotrimers or heterotrimers. Homotrimeric collagens are formed from three identical α-chains, e.g., [α1(III)]_3_ for type III collagen. Heterotrimeric collagens contain different α-chains of the same collagen type, like [α1(I)][α2(I)]_2_ for type I collagen. Some collagens can also occur with various α-chain compositions. An example is type V collagen formed by combinations of three different α-chains, namely [α1(V)][α2(V)]_2_, [α1(V)][α2(V)][α3(V)], or [α1(V)]_3_ [43]. Type I collagen is the most abundant and best described of all collagens. Type I collagen provides a basis for the mechanical properties of many biological materials and makes the most significant part of collagen fibrils of skin, tendons and ligaments, veins and arteries, organs, and bone [41,43].

### 2.1. The Primary Structure

**Figure 1:**
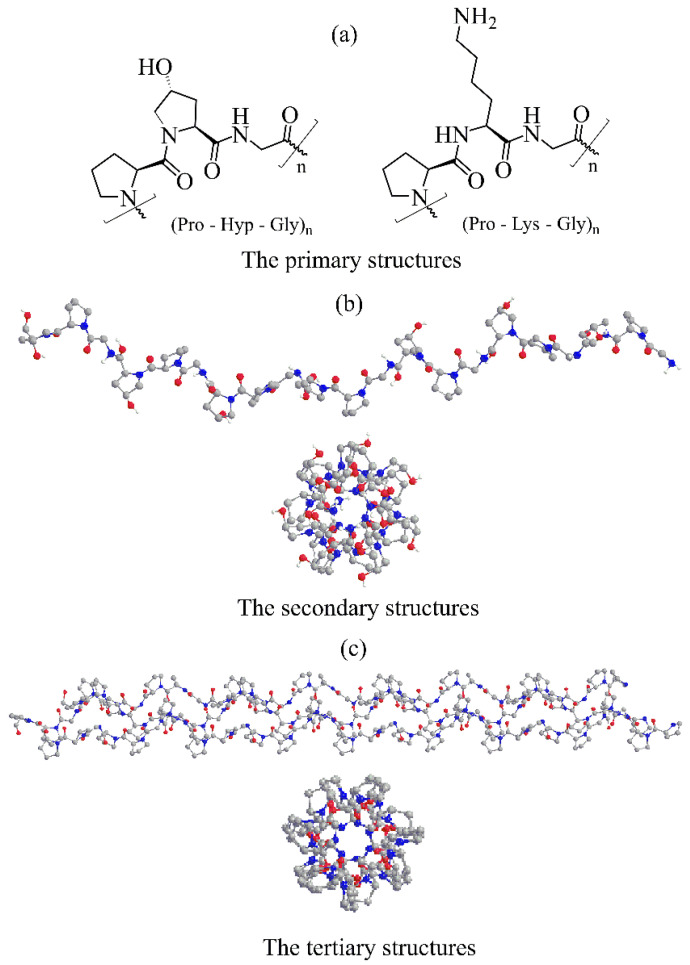
Illustration of collagens molecular structures. (**a**) Schematic representation of the most abundant collagen Pro-Hyp-Gly and Pro-Lys-Gly triplets. (**b**) Crystal structure of a collagen model (Gly-Pro-Hyp)_10_ single chain helix (PDB entry 4OY5, ref. [45]). (**c**) The collagen triple helix of the (Pro-Pro-Gly)_10_ model (PDB entry 1K6F, ref. [46]). Crystal structures are depicted in colored ball-and-stick representations.

Collagens consist of three α-polypeptide chains (Figure 1). The primary structure of polypeptide chains, termed α-chains, affects collagen structural and physiological properties at all hierarchical levels. The defining motif of polypeptide chains is a repeating Xaa-Yaa-Gly sequence presented in all collagen types (Figure 1a). The folding of the three chains requires that Gly is every third residue in triplets. The remaining two amino acids, Xaa and Yaa, are often (*2S*)-proline (Pro, 28%) and (*2S,4R*)-4-hydroxyproline (Hyp, 38%) but can also be any other amino acid, except glycine [30,44] (Figure 1a). As a consequence, the Pro-Hyp-Gly moiety is the most frequent of all possible triplets in collagen (10.8%). There is also a high amount of lysine (Lys) residues in position Yaa (10.5%), reflected in the occurrence of the tripeptide Pro-Lys-Gly of 2.7% [44]. The presence of lysine residue is critical for the structure and behavior of collagen due to its role in post-translational modifications. Each polypeptide chain contains 662 to 3152 amino acids, depending on the collagen type [9].

### 2.2. The Secondary Structure

In all collagens, the polypeptide chain adopts a polyproline II-type left-handed helical conformation [47] with all peptide bonds in the trans conformation [30]. The high-resolution crystal structure of the collagen peptide model (Pro-Hyp-Gly)_10_ [45] revealed that (Figure 1b) the proline rings could adopt two ring conformers, while the hydroxyproline rings prefer only one ring conformation. Hydrogen bonds between backbone amine groups and carbonyl groups of amino acids are essential factors stabilizing the left-handed helix. In collagens, α-chains contain at least one collagenous domain and non-collagenous domains. Both ends of the α-chain, N-terminus and C-terminus are non-collagenous domains.

### 2.3. The Tertiary Structure

Three parallel α-helical chains supercoil around the central axis to form a right-handed triple helix (Figure 1c). Due to difficulties in investigating collagens at the atomic level, detailed information about the triple helix structure was obtained using various collagen peptide models [40], showing that the triple helix conformation is close to the 7/2 helix. The recently solved high-resolution crystal structure of the (Pro-Hyp-Gly)_10_ collagen model [45] at 0.98 Å resolution confirmed the 7/2 helix with the helical pitch corresponding to the length of three amino acids. It provided structural details about the α-chain at the atomic level. This tertiary structure, called tropocollagen, is stabilized by inter-chain hydrogen bonds, and three chains are staggered by one residue relative to each other. The tropocollagen can develop a straight or kinked rope-like rod shape. However, this rod can have a kink or flexible interruption depending on (Xaa-Yaa-Gly)_n_ sequence. Crucial for the helix stability is the presence of glycine that occupies the position in the center of the triple helix. A single replacement of Gly with other amino acids can destabilize the triple helix [30]. The more bulky side chains of the other two amino acids point outward and almost perpendicular to the helix axis. The average molecular weight of the tropocollagen is 300 kDa and <300 nm long with a diameter of 1–2 nm [30]. The collagen triple helix binds to various cell surface receptors such as integrins, discoidin domain receptors, glycoprotein VI, leukocyte-associated IG-like receptor-1, and members of the mannose receptor family [48]. These interactions regulate cell behavior, and collagen gene mutations are associated with various connective tissue diseases [32].

### 2.4. The Quarternary Structure

Tropocollagens self-associate into intermediate-size fibrils called microfibrils [49]. Each microfibril contains five tropocollagen molecules. In microfibrils, tropocollagens are linked end-to-end in a line and parallel but staggered alignment [50]. There is a space between adjacent molecules about 65–67 nm and a distance of 40 nm between two 300 nm long consecutive molecules [51]. The covalent intramolecular and intermolecular cross-linking between lysine and histidine of the C-terminus or N-terminus of tropocollagen molecules is responsible for the stability of insoluble microfibrils [52].

### 2.5. Supramolecular Assemblies

Various classes of collagen structures, such as fibrils, networks, filaments, and transmembrane collagenous domains, are present in the ECM. The following will discuss fibril type I collagen as the principal component of all fibrous tissues except cartilage. After the formation of microfibrils, collagen fibrillogenesis continues by further self-assembling several microfibrils into collagen fibrils with a rope-like structure [53]. The length of collagen fibrils is up to 1 μm with a diameter of up to 500 nm. Thus, the elongation from the collagen triple helix (<2 nm diameter, 300 nm length) to the fibril (500 nm, 1 mm) during fibrillogenesis is remarkable on its scale. In the next step of the hierarchical building process, several thousand (∼4000) collagen fibrils can twist or pack together to form a collagen fiber. Finally, bunches of collagen fibers interact with one another to create the basic organizational structure of the organism with a three-dimensional network [41,49,52].

**Figure 2:**
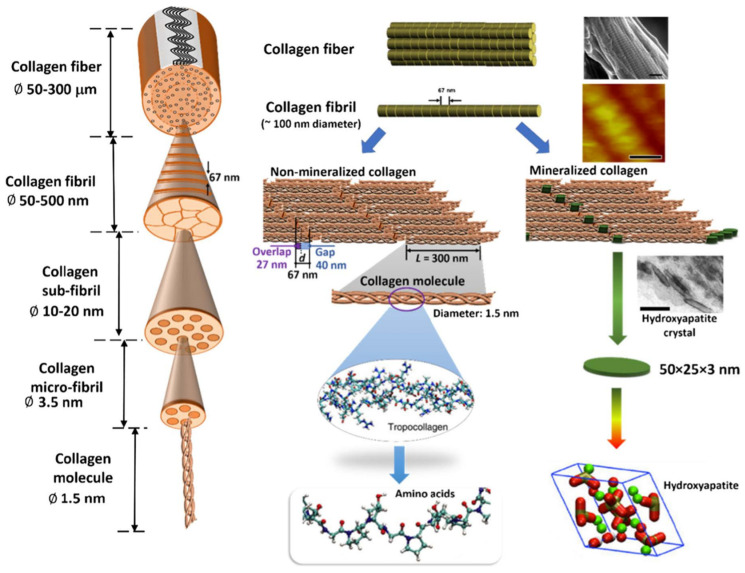
Hierarchical structure of a collagen fiber. The left-hand side shows the hierarchy of the collagen fiber with length-scales. A more detailed illustration of each hierarchy level is explained for non-mineralized and mineralized collagen fibers on the right-hand side. Each collagen molecule comprises three peptide chains forming the ≈300 nm long triple helical collagen molecule. Collections of collagen molecules aggregate both in lateral and longitudinal directions to form fibrils. Reprinted with permission from [41]. Copyright 2019 with permission from Elsevier.

In vivo, collagen protein materials are organized into various supramolecular structures hierarchically, forming high-order assemblies [49,52]. Figure 2 shows an overview of the hierarchy in the structure with its length scales [41]. Collagens from different groups interact with collagen from other groups and with non-collagenous proteins and, together with their abundance, modulate the higher order of tissue structures. They play vital roles in tissue development, growth, repair, and remodeling. On the right hand, Figure 2 illustrates steps in forming non-mineralized and mineralized collagen fibers at these different levels. The formation of nanoscale hydroxyapatite (HAP) crystals is a multistep process (biomineralization) in which water within the collagen matrix is replaced by calcium and phosphate complexes. These complexes are precursors of HAP, which fills the gap regions in the collagen fibrils in hard tissues such as bone and teeth [41,54].

## 3. Post-Translation Modifications of Collagen

**Figure 3:**
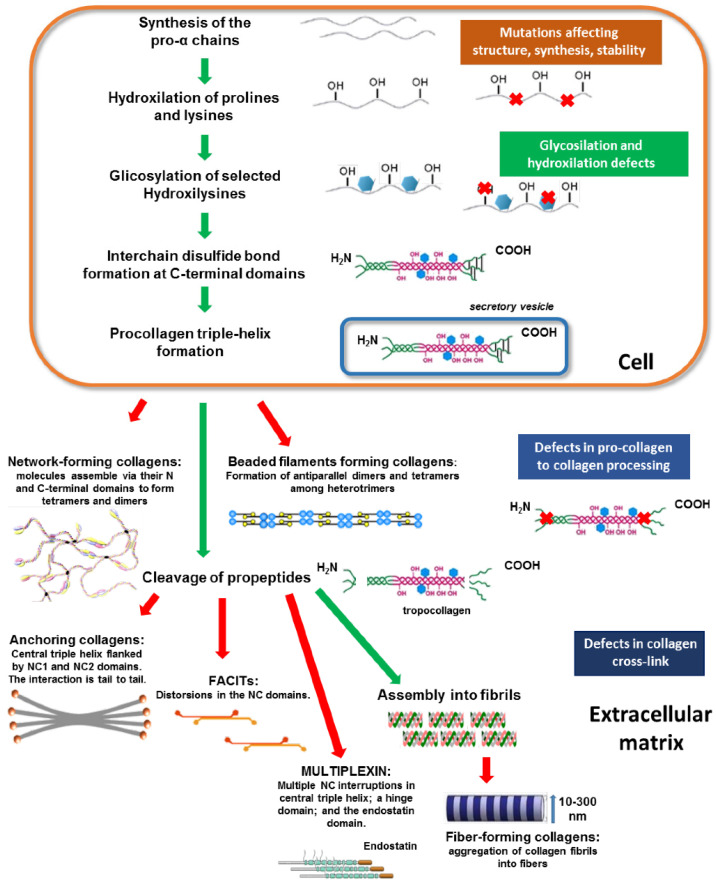
Schematic representation of collagen biosynthesis steps. The main biosynthesis steps of various collagen types are indicated. Green arrows highlight the contiguous processing steps, whereas red arrows indicate the final phase of collagen assembly into the different structural conformations. Colored boxes on the right indicate potential alterations occurring during collagen processing at various steps, thus causing abnormalities in the structure and assembly. Abbreviations: NC, non-collagenous domain. Reprinted with permission from [14]. Copyright 2018 with permission from MDPI.

Collagen biosynthesis, schematically described in Figure 3, assembly and maturation are multistep processes that occur intracellularly and extracellularly and have been discussed in several papers recently [11,38,43,55,56]. Therefore, this review will focus on post-translational modifications critical to collagen’s structure and biological functions. Collagen biosynthesis commences within the nucleus of cells by the synthesis of the single polypeptide pro-α-chain called the pre-procollagen chain, which contains a collagenous α-chain domain and the N- and C-terminal domains. After transcription, the pre-procollagen chain is translocated to the rear endoplasmic reticulum (RER). In the RER, the signaling sequence at its N-terminal domain is cleaved, and post-translational modifications (PTM) are carried out.

Intracellular and extracellular PTMs represent critical steps of collagen biosynthesis that influence collagen’s structure and functionality [32,55,57,58,59,60,61]. Before triple helix formation, the α-chain is stabilized by intra-chain disulfide bonds, and an *N*-glycan is transferred to an asparagine residue in the C-terminal propeptide domain. The principal intracellular PTMs of polypeptide α-chains include the hydroxylation of selected proline and lysine residues followed by the *O*-glycosylation of specific hydroxylysine residues required for proper folding and assemble into a right-handed triple helix to form pro-collagens. Enzymes involved in these modifications are galactosyltransferases and glucosyltransferases [62]. In extracellular space, lysine and hydroxylysine are oxidatively deaminated, leading to reactive aldehydic residues that subsequently form intra and intermolecular covalent cross-linkages influencing the functions of fibrils [58].

In the following events, three pro-α-chains begin to associate at the C-domain and propagate toward the N-terminus, becoming a pro-collagen molecule. The triple helix formation prevents further PTM, and pro-collagen molecules are secreted into extracellular space, where N- and C-domains are cleaved, leading to tropocollagens. Finally, tropocollagens self-assemble into various supramolecular structures for their function depending on the collagen types (Figure 3). 

## 4. Hydroxylation of Proline and Lysine Residues

**Figure 4:**
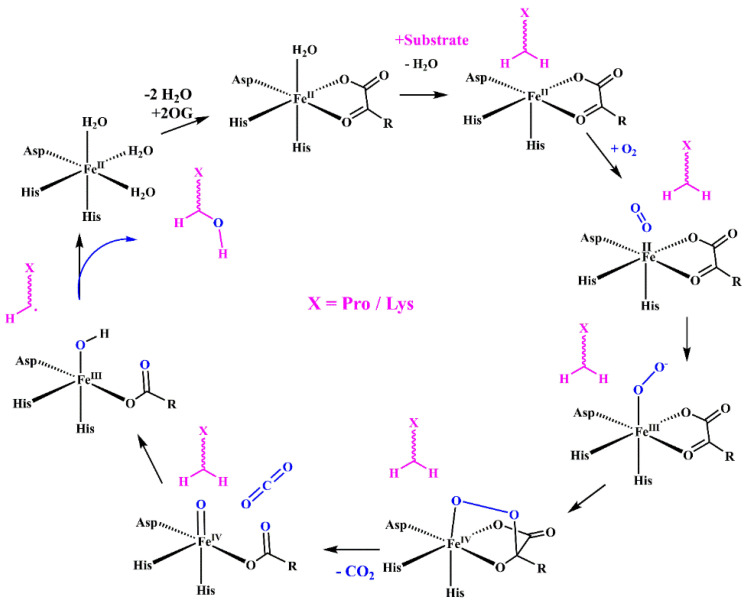
Mechanism of the Fe(II)- and 2-OG-dependent dioxygenases including P4H, P3H, and LH hydroxylases. The enzyme active site consists of a ferrous iron ion coordinated by two His residues and one Asp residue. The catalytic reaction consists of several consecutive steps: binding of 2-OG displacing two water molecules; the substrate binding replaces a third water molecule; binding oxygen and forming an anionic intermediate; the formation of a cyclic peroxide molecule by attacks the ketone of 2-OG; the decomposition of intermediate and the formation of the Fe(IV)oxo species; the abstractions of hydrogen from the substrate; and the reaction of substrate radical with the Fe(III)−OH complex to form the hydroxylated substrate.

In the procollagen triple helix, repetitive Xaa-Yaa-Gly sequences contain numerous hydroxyproline and hydroxylysine residues in the Xaa and Yaa positions. Their presence is a consequence of enzymatic hydroxylation by three collagen-specific enzymes, namely prolyl 4-hydroxylases (P4H), prolyl 3-hydroxylases (P3H), and lysyl hydroxylases (LH) that hydroxylate numerous proline and lysine residues into hydroxyproline (Hyp) and hydroxylysine (Hyl) throughout PTMs [32,34,57,59,61,63]. These enzymes are a large and diverse superfamily of 2-oxoglutarate-dependent oxygenases (2OGXs) that catalyze various oxidative reactions [64,65,66]. The catalytic mechanism of these hydroxylases is similar and requires Fe^2+^ as a cofactor and molecular oxygen (O_2_), 2-oxoglutarate (2OG), and ascorbate as co-substrates (Figure 4).

### 4.1. Prolyl 4-Hydroxylase

**Figure 5:**
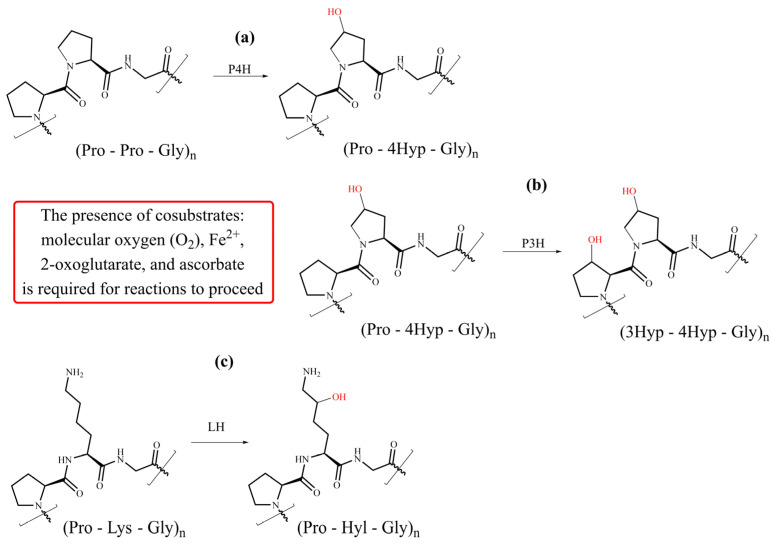
Schematic representation of collagen-specific PTMs—hydroxylation of proline and lysine residues with (**a**) P4H (prolyl 4-hydroxylases), (**b**) P3H (prolyl 3-hydroxylases), and (**c**) LH (lysyl hydroxylases).

Collagen prolyl 4-hydroxylase (P4H, EC 1.14.11.2) is the enzyme crucial for collagen biosynthesis. P4H exists as the heterotetramer α2β2 formed from α and β subunits. The tetrameric assembly is required for the stability and total activity of the enzyme. P4H catalyzes proline 4-hydroxylation at the Yaa position in various Xaa-Pro-Gly sequences of the α-chains (Figure 5a) with different efficiency [67,68,69]. The 4-proline hydroxylation is critical for the thermal stability of collagen at physiological temperatures. Proline 4-hydroxylation is essential for assembling procollagen chains into triple helices through hydrogen bonding and also influences interactions between integrins and collagens [70]. For collagen P4H, several isoenzymes have been identified. Three isoforms of the α subunit, α(I), α(II), and α(III), were identified with α(I) prevailing. The α subunit forms the catalytic domain responsible for substrate recognition and enzymatic activity. The β subunit functions as protein disulfide isomerase (PDI) [59,69,71]. Following decades of unsuccessful attempts to solve the high-resolution structure of P4H, recently, the crystal structure of the catalytic domain of human collagen P4H-II of the CAT domain complexed with the intact β/PDI subunit has been solved at a 3.8 Å resolution [72].

Also, structures of several other P4H enzymes and functional domains of the subunit and PDI have been determined [68,72,73,74,75,76,77,78]. The crystal structure of P4H has been complemented by a quantum mechanics/molecular mechanics molecular (QM/MM) modeling study on how Glu127 and Arg161 mutations affect substrate binding [79]. P4H is crucial in collagen synthesis and has been associated with fibrotic diseases, hypoxia, and cancer [80]. Therefore, these data have provided valuable information for the rational design of potential therapeutic for fibrosis and cancer [57].

### 4.2. Prolyl 3-Hydroxylase

Collagen prolyl 3-hydroxylase (P3H, EC 1.14.11.7) catalyzes the hydroxylation of proline residue in position 3 (Figure 5b). The substrate for a P3H action is the (Pro-4Hyp-Gly) sequence [57,59,63,81]. Not all proline residues in such moieties are hydroxylated; the extent of hydroxylation depends on the collagen type and is tissue-specific. Three isoforms exist in humans, namely P3H1, P3H2, and P3H3. All have 2OGX domain homologous with P4H and proceed via a similar reaction mechanism [81]. An active P3H1 forms a complex with the cartilage-associated protein (CRTAP) and the peptidyl-prolyl *cis-trans* isomerase cyclophilin B (CYPB) in 1:1:1 stochiometry [81]. Such a complex is not needed for the functioning of P3H2 and P3H3. The P3H1 isoform occurs in tissues full of fibrillar collagens, whereas P3H2 is mainly expressed in tissues rich in basement membranes, such as the kidney [82,83]. The function of P3H is not fully understood, although inactivating P3H1 causes a severe, recessive form of osteogenesis imperfecta characterized by skeletal deformities and bone fragility [84,85]. It was suggested that 3-proline hydroxylation plays an essential biological function by influencing the ordered self-assembly of collagen supramolecular structures [86,87].

### 4.3. Lysyl Hydroxylases

Collagen lysyl hydroxylase (LH, EC.1.14.11.4) catalyzes the hydroxylation of lysine residues (Figure 5c) in ER and leads to 5-hydroxylysine (Hyl). There are three isoforms of LH in humans: LH1, LH2, and LH3 encoded by *PLOD1*, *PLOD2*, and *PLOD3* genes, respectively. A catalytic mechanism of LH is similar to that described for propyl hydroxylases (Figure 4). A formation of LH dimers is required for their hydroxylation activity [88,89]. Hydroxylation precedes two fundamental modifications of collagen: glycosylation and covalent cross-linking [57,58,59,60]. The hydroxylation of lysine residues in the Xaa-Lys-Gly sequence dominates, although Xaa-Lys-Al and Xaa-Lys-Ser moieties are also hydroxylated in the telopeptide domain. Notably, a lysine residue in the Xaa position is not hydroxylated. Compared with proline hydroxylation, the extent of lysine hydroxylation is more variable, depending on the tissues and physiological conditions, and telopeptide and helical domains are hydrolyzed differently [60]. While the LH1 hydroxylates lysines are located in the helical domain, the LH2 is solely responsible for hydroxylation in the telopeptide domain [25,90,91].

Recent biochemical and homology modeling studies revealed that LH1 and LH2 exhibit, before unobserved, galactosylhydroxylysyl glucosyltransferase (GlcGalHylT) activity, thus being bifunctional enzymes [92]. The LH3 is a multifunctional enzyme, and it was assumed that it bears three different catalytic activities, namely lysine hydroxylase (LH), hydroxylysyl galactosyltransferase (GalHylT), and galactosylhydroxylysyl glucosyltransferase [88,93,94,95,96]. However, recent experimental findings indicate that the primary function of LH3 is the final step of glycosylation, that is, the transfer of the glucopyranose residue to galactohydroxylated lysine [97,98,99]. 

## 5. Oxidation of Lysyl Residues

**Figure 6:**
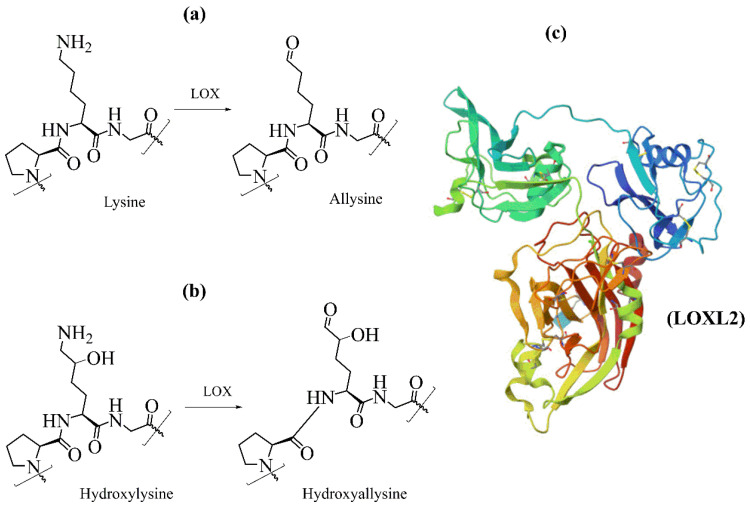
Schematic representation of collagen-specific oxidative deamination oxidation by lysyl oxidase (LOX) of (**a**) lysine and (**b**) hydroxylysine and (**c**) the 3D structure of LOXL2, PDB ID code 5ZE3 [105].

After secretion into extracellular space, some telopeptide hydroxylated lysine residues are catalytically oxidized by enzymes lysyl oxidases. The oxidative deamination of lysine residues is a prerequisite for the spontaneous formation of covalent cross-links that stabilize developing fibrils. The lysyl oxidases family comprises five highly homologous members: lysyl oxidase (LOX, EC 1.4.3.13), lysyl oxidase like-1 (LOXL1), lysyl oxidase like-2 (LOXL2), lysyl oxidase like-3 (LOXL3), and lysyl oxidase like-4 (LOXL4) [100,101,102]. Lysyl oxidases are copper-dependent enzymes that catalyze the oxidative deamination of Lys and Hyl residues in telopeptide domains of collagen (Figure 6), leading to their aldehydes [58]. The catalytic domain of all LOX family members is highly conserved; therefore, they are assumed to have very similar catalytic properties [103]. This reaction is the first step in covalent collagen crosslinking in ECM [34,42,60,104]. The catalytic site of LOX contains copper ion (Cu^2+^), and the reaction requires the covalently integrated cofactor lysyl tyrosylquinone (LTQ). Recently, the crystal structure of human lysyl oxidase-like 2 (hLOXL2) at a 2.4-Å resolution [105], and this structure has been used as the template to generate a homology 3D model of LOX [106]. 

**Figure 7:**
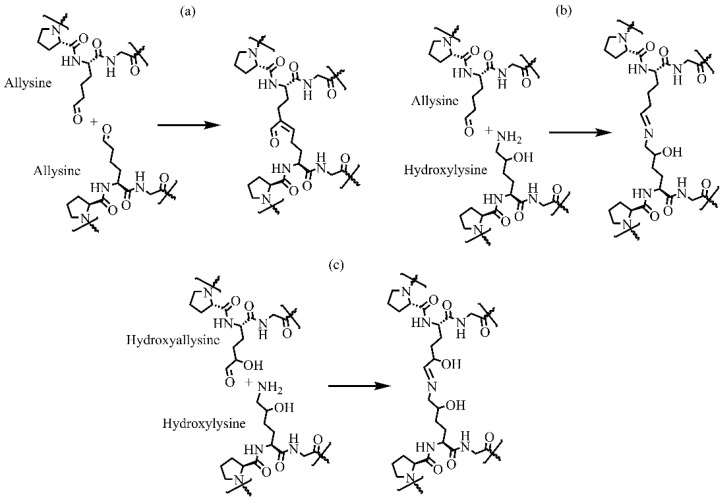
Schematic representation of three major physiological intramolecular cross-links by condensation between (**a**) two allysines forming an aldol condensation product, (**b**) allysine and hydroxylysine forming dehydro-hydroxylysinonorleucine, and (**c**) hydroxyallysine and hydroxylysine forming dehydro-hydroxylysinohydroxynorleucine.

The resulting telopeptidyl aldehydes spontaneously react with other aldehydes or free lysine and hydroxylysine in a series of condensation reactions, generating various intra- and intermolecular cross-links that are crucial for biomechanical properties of collagens. Collagens have three major cross-linking pathways (Figure 7) [60]. Lysine-based cross-linking occurs mainly in adult skin, cornea, and sclera. In contrast, hydroxylysine-based cross-links prevail in bone, cartilage, ligament, most tendons, embryonic skin, and internal connective tissues of the body. In humans, cross-links decreases with age [34].

## 6. Glycosylation of Collagens

Collagens belong to the group of human secretory proteins that are modified by adding carbohydrate molecules using two different mechanisms: enzymatic (glycosylation) and non-enzymatic (glycation). Due to its association with aging and diabetes, glycation has become one of the most studied processes. Many papers have been published, and several reviews discussed the progress in understanding glycation’s mechanism and biological consequences [107,108,109,110,111,112]. It is beyond the scope of this review to describe glycation in detail and reference available literature. Therefore, only a very brief overview of glycation will be given. Less attention has been devoted to the second mechanism, glycosylation, than glycation [21,113]. The aim is to provide the reader with an overview of the recent progress in this area.

### 6.1. Glycation

The reaction of glucose in the aldehyde form (and several other monosaccharides presented in the blood) with lysine and arginine residues in proteins is called glycation. The formed glucosyl-lysine intermediates are stabilized via further reactions, including Schiff bases and Amadori products. This leads to complex advanced glycation end products (AGEs), including intra and intermolecular cross-links [107,110,114]. Glycation is a slow process since the initial reaction of glucose with lysine requires the conformational change in the sugar from the dominant cyclic form to the open-chain form. Collagens are very long-lived proteins and, therefore, are susceptible to glycation.

The physiological cross-linking of collagen is carried out enzymatically by lysyl oxidases (LOX), located at the ends of tropocollagens, modulating optimal properties and stabilizing the different supramolecular structures of collagen [104]. On the contrary, glycation triggers pathological cross-linking located randomly in the helical regions of fibrous collagens that increases with age. It has been shown that glycation might significantly impair LOX-controlled cross-linking [115]. AGEs cross-linking alters collagen physical properties (fibril stiffness) and influences intermolecular interactions with other ECM components, greatly influencing collagen’s biological function. Collagens are relevant ligands of integrins [116], and their altered fibril structure likely affects the binding to integrins and thus impairs integrin outside–in and inside–out signaling. Although the glycation of collagen plays an essential role in aging and diabetes [107,108,110], it has also been implicated in various severe diseases [107,109], among others, in vascular diseases [109,117,118], and cancer [119,120,121,122].

### 6.2. Enzymatic Glycosylation

Glycosylation is the most abundant and perhaps the most structurally diverse post-translational modification of proteins consisting of several sequential steps in the endoplasmic reticulum and Golgi apparatus that involve numerous glycosyltransferases and glycosidases [123,124,125]. Glycosylation proceeds through several different pathways that reflect the presence of glycosyltransferases (GTs) and their substrates in the cell. Glycosyltransferases synthesize glycan structures by catalyzing the transfer of monosaccharide residue from an activated donor nucleotide sugar to the hydroxyl group of an acceptor. Based on the similarity of their primary sequences, GTs were clustered into 117 families [126,127] and registered in the Carbohydrate Active Enzyme Database (CAZy, available at http://www.cazy.org).

An analysis of known crystal structures revealed the existence of three general folds for GTs: GT-A, GT-B, and GT-C [128,129,130,131]. From a mechanical point of view, the catalytic reaction is quite complex. It requires the presence of a donor nucleotide sugar in the adequate micro-concentration and an acting glycosyltransferase in a Golgi compartment. Also, the working glycosyltransferase must be sufficiently expressed in the productive conformation that accommodates the donor and acceptor in the reaction Michaelis complex [124]. In sugar-nucleotides, the sugar residue is linked to UDP by the α-glycosidic linkage (α-anomer), and an attachment of the transferred monosaccharide residue to an acceptor can be achieved by the α- or β-glycosidic bond. Based on the resulting linkage, glycosyltransferases are classified into retaining or inverting enzymes [130]. 

The produced glycans show enormous structural diversity [132], allowing for adjusting their 3D structure to biological function [131,133]. In the case of collagens, two different glycans have been observed: the highly conserved *N*-glycan in the C-terminal propeptide domain and *O*-glycans on hydroxylated lysine residues. Glycosylation on collagens occurs in the Golgi apparatus and ER before triple helix formation.

#### 6.2.1. *N*-Glycan

The presence of *N*-glycan on collagen has been observed in the C-terminal propeptide domain [134,135,136,137,138,139,140,141,142]. These studies also revealed that glycan is a high mannose oligosaccharide containing two *N*-acetylglucosamine (GlcNAc) and six to nine mannose residues. *N*-glycosylation is a common PTM occurring in most secretory proteins [143,144,145,146]. The glycosylation of proteins starts in ER with the biosynthesis of oligosaccharide GlcNAc_2_Man_9_Glc_3_ linked to dolichol phosphate. It continues by transferring oligosaccharide en bloc to protein by forming a β-1-*N*-linkage between GlcNAc and the Asn side chain. Then, the repertoire of glycosidases and glycosyltransferases in the ER and Golgi apparatus synthesize a massive variety of *N*-glycans that differ in composition. 

**Figure 8:**
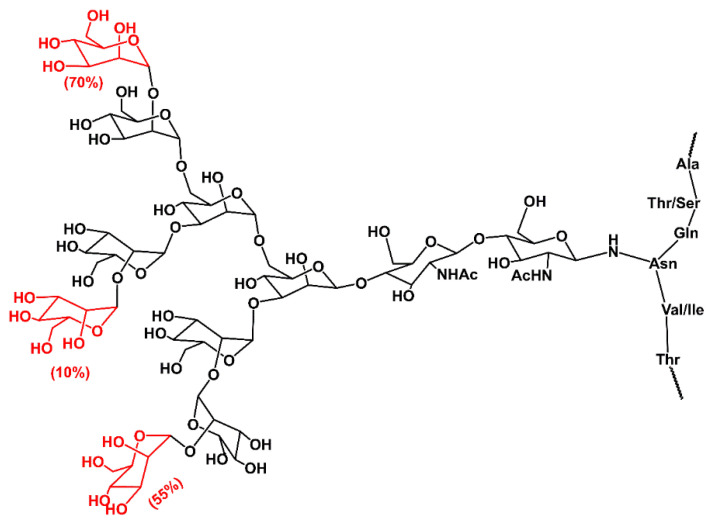
Schematic representation of *N*-glycan structure from C-terminal propeptide domain of chick embryo procollagen I, as determined by high-resolution spectra 500 MHz ^1^Hs-NMR. The percentages refer to the relative occurrence of those mannose residues (shown in red) in a mixture [147].

The structure of collagen *N*-glycan was determined by 500 MHz ^1^H NMR spectroscopy [147]. The isolated and purified glycans were a heterogeneous mixture of high-mannose type glycans [147], as shown in Figure 8. The one carbohydrate unit contains two *N*-acetylglucosamine residues and between six and nine mannose residues (GlcNAc_2_Man_6-9_). The percentages on terminal residues (shown in red) in Figure 8 refer to the relative occurrence of these residues in the mixture. This implies that after the transfer of oligosaccharide GlcNAc_2_Man_9_Glc_3_ from dolichol phosphate to the Asn of collagen C-terminal propeptide, only several glycosidases are involved in the final shaping of the glycan structure. In the ER, the first three glucose residues and one mannose residue are trimmed by α-glucosidases α-Glc-I and α-Glc-II and by the ER α-mannosidase (α-Man), respectively [143,145]. Then, the properly folded collagen with linked glycan Glc_2_Man_8_ is transported to the Golgi apparatus for further processing by Golgi α-mannosidase (α-Man-I), leading to high mannose *N*-glycan GlcNAc_2_Man_6_ [148]. The NMR structure suggests processing of the *N*-glycan at the C-terminal propeptide finishes here. The observed heterogeneity likely reflects the influence of cellular and micro-environmental factors affecting glycosylation efficiency. In contrast to collagens, *N*-glycans maturation in other glycoproteins continues with the concerned action of glycosyltransferases and glycosidases forming branching, elongation, and capping [144,145].

Glycans are integral to protein function and regulate their diverse biological functions in physiological and pathological events by interacting with lectins. The *N*-glycosylation motif is highly conserved within the *C*-terminal domain of fibrillar pro-collagens [134,135,137,140], which suggests that the glycan has an important structural or biological function. However, the C- and N-terminal pro-collagen domains are trimmed as soon as fibrillar pro-collagen is secreted into extracellular space. This suggests that the *N*-glycans have a somewhat more relevant function inside the cell than an extracellular function. Although the function of the *N*-glycan inside the cell is well described (Helenius and Aebi 2001, Helenius and Aebi 2004), the function of the collagen *N*-glycan remains incomplete.

#### 6.2.2. *O*-Glycans

The first mention of the carbohydrate molecules in collagens dates almost 90 years ago, when glucopyranose and galactopyranose in a 1:1 ratio were observed in the collagen of animal skin [149]. In the next two decades, further characterizations of saccharides in collagen were determined, and it was shown that these saccharides are bound to the peptide by a glycosidic linkage [150,151]. Studies on carbohydrate contents in various collagens have revealed two different saccharides on hydroxylysine, namely a disaccharide α-d-glucopyranosyl-(1→2)-β-d-galactopyranose or a monosaccharide β-d-galactopyranose [20], suggesting the action of two enzymes in ER. A comparison of the extent of glycosylation in various collagens showed that basement membrane collagens contain more glycosylated hydroxylysines than fibrillar collagens and that the disaccharide prevails. Two glycosyltransferases involved in the biosynthesis of glycans were isolated from a rat kidney cortex and characterized [152,153,154].

**Figure 9:**
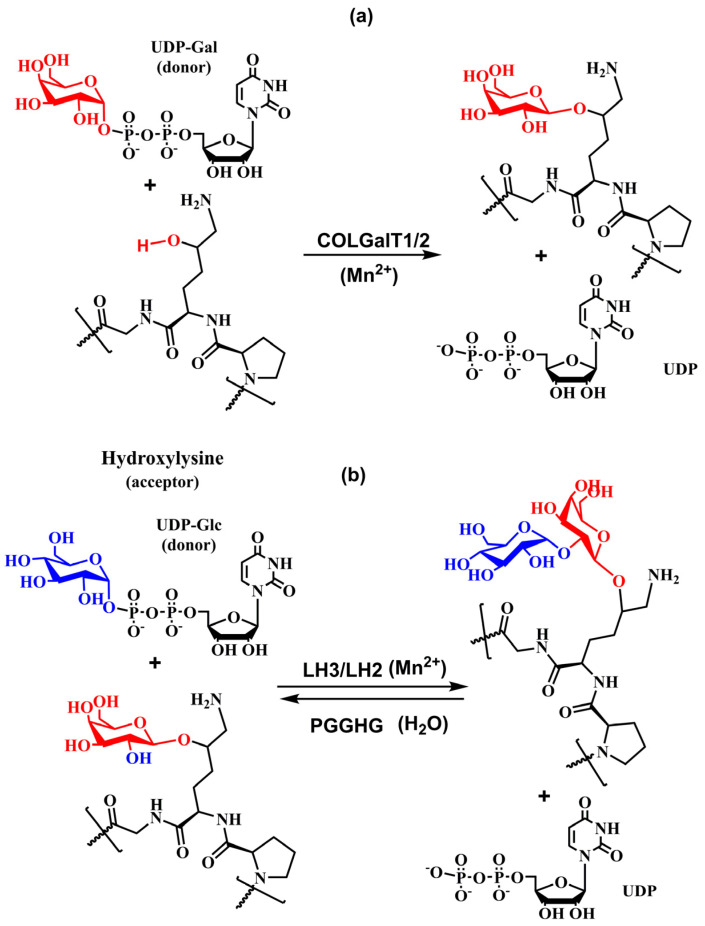
Schematic diagram of the enzymatic reaction catalyzed by (**a**) COLGALT1/2 and (**b**) LH2 and LH3.

The first step of the glycosylation, the addition of β-d-galactopyranose to the hydroxyl group of the hydroxylysine side chain (Figure 9a), is catalyzed by procollagen-galactosyltransferase (UDP-galactose: procollagen-5-hydroxy-l-lysine d-galactosyltransferase, EC 2.4.1.50) that transfers galactose from UDP-galactopyranose to hydroxylysine by the β-linkage [62,97,155]. Then, procollagen glucosyltransferase (UDP-glucose: 5-(d-galactosyloxy)-l-lysine-procollagen d-glucosyltransferase, EC 2.4.1.66) catalyzes transfer glucopyranose via the α-(1→2) linkage to galactosyl-hydroxylysine [62,152,154,156,157,158] (Figure 9b). A free ε-amino group of the hydroxylysine residues is necessary for both of these reactions to carry on [157]. Later, it was realized that these enzymatic activities were also carried out by a multifunctional enzyme lysyl hydroxylase LH3 (LH, E.C. 1.14.11.4) with the primary function catalyzing glucopyranose transfer [88,93,94,95,96,97,98,99]. The number and distribution of glycans differ significantly between and even within the same collagen type. The final glycosylation outcome is influenced by many factors, including the presence of UDP-Gal and UDP-Glc donors, the availability of lysine hydroxylases and glycosyltransferases, cofactors, protein chaperons, and generally on the condition of the ER microenvironment that depends on various physiological and pathological states.

### 6.3. Procollagen Galactosyltransferases

Two isoenzymes, COLGALT1 and COLGALT2 [159,160,161], were identified and encoded by *COLGALT1* (UniProt Q8NBJ5) and *COLGALT2* (UniProt Q8IYK4) genes, respectively [91,162]. The procollagen galactotransferases COLGALT1 and COLGALT2 are transmembrane glycoproteins of family 25 with 622 and 626 amino acids, respectively [97,158]. The COLGALT1 and COLGALT2 have been shown to catalyze the transfer of galactose to procollagen Hyl from the activated donor UDP-Gal [97,158,162]. The catalytic reaction proceeds in the endoplasmic reticulum before forming a triple helix. The two procollagen galactosyltransferases have different tissue distributions. While COLGALT1 is constitutively expressed in all human tissues, COLGALT2 is only expressed at low levels in the nervous system.

Site-directed mutagenesis has been used to examine the presence of the DxD motif in COLGALT1 [162], and three DxD motifs were localized in these enzymes, one in the N-domain and two in the C-domain. In COLGALT1, only one DxD in the C–domain was not required for the galactotransferase activity. Also, it was suggested that the UDP-Gal donor and the metal cofactor bind to the DxD motif in the N-domain [162]. The variable DxD motif is the characteristic feature of enzymes with GT-A fold [124,129,131,163]. Two α/β/α Rossmann-like folds create the active site containing a DxD motif [128]. The enzymes of the GT-A superfamily have two distinct donor and acceptor binding sites with a crucial role played by a disordered loop that changes conformation after binding an acceptor [164]. The kinetics measurements of apparent *K*_m_ of COLGALT1 and COLGALT2 provided values of 13.6 g/liter and 9.8 g/liter for the bovine type I collagen acceptor and 18.77 μM and 33.53 μM for the UDP-Gal donor, respectively [97]. The sequence alignment of both human COLGALT revealed a 63% identity [97], suggesting that the same DxD sequence is responsible for their activity. From a chemical point of view, COLGAT enzymes are the metal-ion-dependent (Mn^2+^) inverting glycosyltransferases, in which the formation of a new glycosidic linkage between anomeric carbon (C1) of galactose from the donor UDP-Gal and 5-oxygen from the acceptor collagen Hyl proceeds with the inversion of stereochemistry at the anomeric carbon C1. Experimental and theoretical calculations support an S_N_2-like direct-displacement mechanism for inverting glycosyltransferases [124,128].

**Figure 10:**
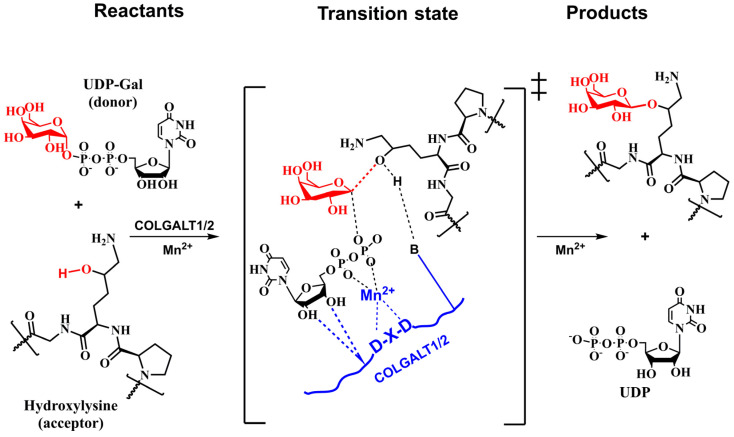
Schematic representation of the tentative SN2-like direct-replacement catalytic mechanism of inverting COLGALT1/2 transferases based on the analogy with inversing glycosyltransferases [124,165].

The analogy with inverting enzymes having GT-A fold leads to the tentative mechanism shown in Figure 10, where the DxD motif binds the bivalent ion (Mn^2+^). The chelating bivalent metal serves as the donor’s active site anchor, which is stabilized by a hydrogen bond between COLGAT amino acids and hydroxyls from ribose. The enzyme also provides a catalytic base that deprotonates attacking hydroxyl from the acceptor for the reaction to proceed [124,130].

**Figure 11:**
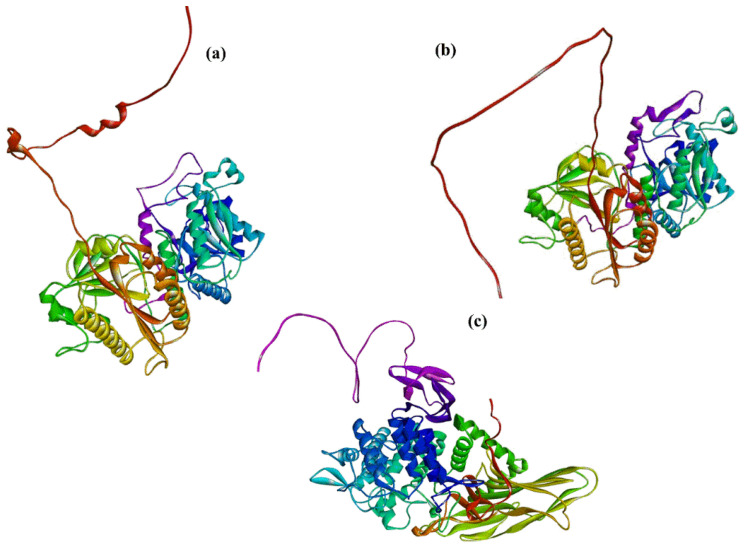
The AlphaFold homology models with per-residue model confidence score pLDDT > 89% of (**a**) COLGALT1 (AF-Q8NBJ-F1), (**b**) COLGALT2 (AF-Q8IYK4-F1), and (**c**) PGGHG (AF-Q32M88-F1).

The structure of COLGAT enzymes has not been solved yet. However, the recently developed deep-learning method AlphaFold [166,167] generated, among others, the homology models of COLGALT1 and COLGALT2, as shown in Figure 11a,b. Both AlphaFold models have a high (>89%) per-residue model confidence score (pLDDT).

Surprisingly, no glucosylation activity has been detected for two galactosyltransferases [97,158]. Conversely, the glucosylation enzymatic activity has been assigned to lysyl hydroxylate LH3 [95]. It was also shown that the sub-cellular locations of LH3 and COLGALT1 in ER are comparable [158].

### 6.4. Lysyl Hydroxylases

For a long time, it was assumed that of three isoforms of LH in humans, LH3 is the only one exhibiting glycosyltransferase activity. However, a recent study [92] discovered glucosyltransferase activity in LH1 and LH2 isoforms, although this activity was higher in the LH2b isoform. The authors used a luciferase assay to follow a release of the reaction product, UDP, from the reaction of the donor UDP-Glc with synthetic amino acids or deglucosylated type IV collagen. In the assay, all LH showed detectable GlcGalHylT activities that were abolished by a mutation of amino acid residues chelating Mn^2+^ [92]. To support these findings, the authors measured the binding affinity of UDP-Glc to LH2 isoforms microscale thermophoresis, which led to the value of *K*_d_ = 10 μM for LH2b. A comparison of domains bearing glycosyltransferase activity has shown a 60% identity between LH3 and LH1 and a 57% sequence identity between LH3 and LH2. The LH1, LH2, and LH3 active sites possess D-X-X-D, E-X-X-D, and D-X-X-D motifs, respectively. They also generated a 3D homology model of LH2b using the LH3 crystal structure (PDB 6FXT) as the template [25]. They suggested that a recognized loop may enhance the GlcGalHylT activity by serving as the active site cap in LH2b.

It was suggested that the *N*-terminal of LH3 shows glycosyltransferase activity, whereas the C-terminal exhibits lysyl hydroxylase activity [88,94,95,96]. The *PLOD3* gene encodes the multifunctional enzyme LH3. The loss of glycosylation activity does not impair the lysyl hydroxylase activity. The lysyl hydroxylase activity depends on the dimerization of LH, whereas its dimerization is not required for the glycosylation activities [89,168]. Although LH3 catalyzes transfers of both the galactose and glucose residues to procollagen hydroxylated lysine, the transfer of glucose is more efficient [25,169]. The catalytic mechanism of the LH3 is fascinating. While in the galactosylation reaction, LH3 functions as the inversing GT (Figure 10); in glucosylation, the catalytic reaction proceeds via the retaining mechanism with the retention of stereochemistry at the anomeric carbon C1 of glucose.

On the other hand, both reactions are metal-ion-dependent. A kinetics experiment of glucosylation reaction (Myllyila, 1976) revealed an ordered mechanism with Mn^2+^ binding first, then binding UDP-Glc, and the acceptor is the last entering the active site. The product release proceeds the opposite way; the first leaves glucosylated procollagen, then UDP and Mn^2+^, although Mn^2+^ may not leave during each catalytic cycle. Although the primary functional location of LH3 is in the ER, LH3 was also found secreted and active in extracellular space [170].

**Figure 12:**
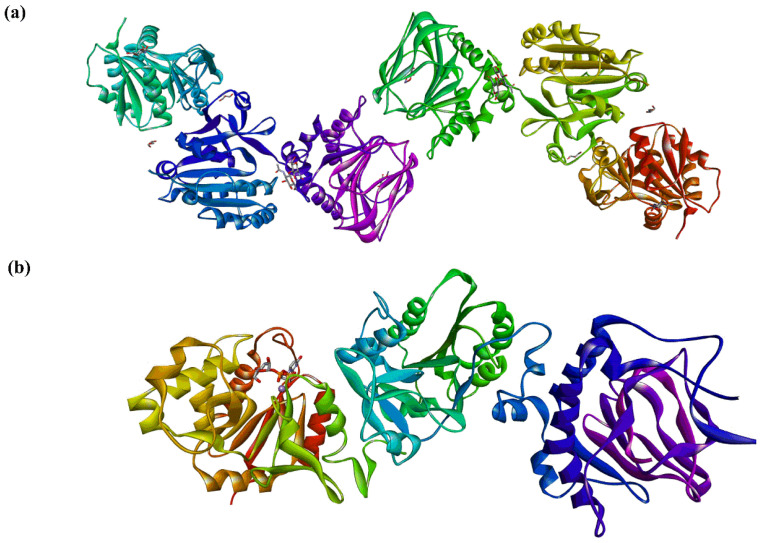
The 3D structure of human LH3 [25]: (**a**) tail-to-tail dimer observed in the crystal structure (PDB ID code 6FXK), and (**b**) three domains of the crystal structure of human LH3, the left is the catalytic glucosyltransferase domain; only the bound donor UDP-Glc and metal cofactor Mn^2+^ are shown (PDB ID code 8ONE).

Recently, in a series of papers, the crystal structures of full-length human LH3 in complex with metal bivalent ion (Mn^2+^ and Fe^2+)^, donors (UDP-Glc and UDP-Gal), and various sugar-nucleotide analogs (PDB entries: 6FXK, 6FXM, 6FXR, 6FXT, 6FXX, 6FXY [25], 6TE3, 6TEC, 6TES, 6TEU, 6TEX, 6TEZ, [28] 6WFV [26], and 8ONE [27]) were solved. Crystal and solution data revealed for LH3 an elongated tail-to-tail homodimer structure of 200 kD and 20 nm long [25]. Two LH3 monomers are connected by two C-terminal domains and stabilized by hydrophobic contacts and electrostatic interactions between amino acids at the interface (Figure 12). It was observed that while the LH activity is associated with the dimer, GT activities were undisturbed in the LH3 monomer, thus supporting previously published data [89,168]. The solved crystal structure [25] revealed that LH3 comprises three domains. The first two N-terminal domains show Rossmann-fold resembling GT-A fold architecture of GTs [128,129,130]. The third C-terminal domain exhibits a double-stranded β-helix fold observed in dioxygenases [171]. The glycosyltransferase and LH activities were located in two 80 Å separated catalytic sites in the N-terminal and C-domain.

The solved LH3 structures permitted the localization of the glycosylation active site and identified crucial amino acids involved in binding the bivalent metal ion and stabilizing the donor. In the complex of LH3 with UDP-Glc and Mn^2+^, two aspartate residues, 112 and 115; histidine 253; and two water molecules chelate metal. Co-crystallization with donor either UDP-Gal or UDP-Glc led to the recognition of the LH3-specific D-X-X-D (Asp112-Ser113-Tyr114-Asp115), where Ser113 and Tyr 114 together with Val44 interact with ribose hydroxyls. Tyr 114 and Trp75 residues sandwich the uracil residue and stabilize its position by π–π interactions along with hydrogen bonds between Thr46 and uracil residue [25]. In the case of LH3, similarly to other GTs [128], the electron density of monosaccharide residue was weak, likely due to several Glc (or Gal) conformations, and it was insufficient to model these residues. Interestingly, the crystal structures of LH3 in complexes with UDP-Glc and UDP-Gal showed no difference.

The question is how the structurally identical active site can orchestrate two different catalytic reactions (inverting vs. retaining, Glc vs. Gal, and different acceptors) requiring different micro-environments. However, it was recently claimed that LH3 is not responsible for the transfer of Gal to Hyl, and the primary function of LH3 is glucose transfer [27,98,172]. Another hypothetical explanation might be that binding different acceptors will reshape the active side differently for each glycosylation reaction. The donor binding structuralizes the flexible loop Hyl72-Gly87 and changes the conformation of Trp145. The mutation of Trp145 into alanine completely abolished glucosylation [27,28], supporting a “gating” role in the active site suggested for Trp145 [25]. Glu141 was indicated as a potential catalytic nucleophile from amino acids in the neighborhood of a nucleotide sugar in the active site since the Glu141Ala mutation completely blocked the sugar transfer [27,28]. However, the distance between Glu141 oxygen and the anomeric oxygen in the LH3 crystal structure (PDB entry 8ONE) [28] (>7 Å) seems to be too long for this function. 

Compared to the inverting [173] GTs, the catalytic mechanism of retaining GTs is more complex, and three different mechanisms were discussed [174]. A classical double-displacement mechanism involving a covalent glycosyl–enzyme intermediate was proposed for α-1,3-galactosyltransferase [175,176] and for A and B α-1,3-galactosyltransferases [177,178]. The one-step front-face reaction (internal return-like or S_N_i) mechanism was proposed for the retaining GTs LgtC [179,180,181] and UDP-GalNAc polypeptide: GalNAc transferase-T2 [173] and α-1,3-galactosyltransferase [182,183]. Also, a slightly modified S_N_i mechanism by including a short-life oxocarbenium ion intermediate, the step-wise front-face reaction mechanism (step-wise S_N_i or ion-pair), was proposed [130] and used for the trehalose-6-phosphate synthase [184], α-1,2-mannosyltransferase [185], and the polypeptide UDPGalNAc transferase [186,187,188]. GTs utilize the S_N_i-like mechanism without a properly located nucleophile in the neighborhood of the anomeric carbon at the transferred sugar donor.

**Figure 13:**
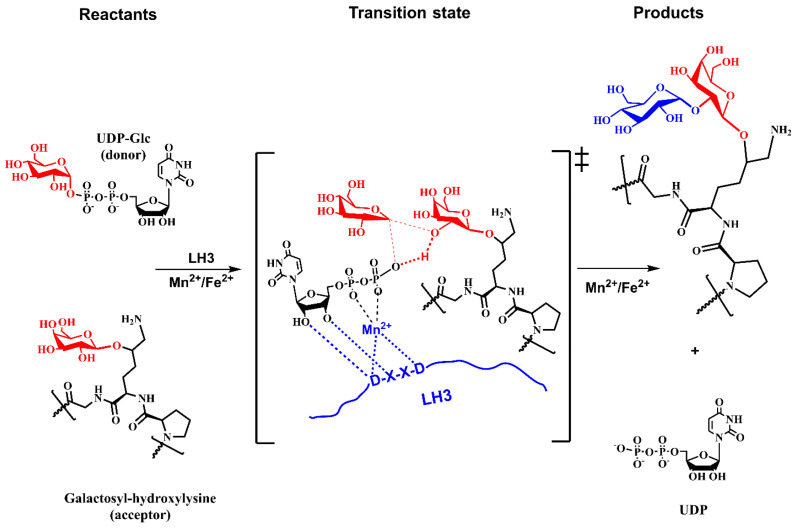
Schematic representation of the tentative S_N_i-like mechanism of the retaining glucosyltransferase LH3 based on the analogy with the LgtC mechanism [179,180].

Experimental data characterized the mechanism as an ordered [159] and concerned mechanism [28] and, together with lacking a properly located nucleophile [27,28], indicated that LH3 might utilize the S_N_i-like mechanism for glucosylation of galactosylated-Hyl. The analogy with the proposed LgtC mechanism [179,180] is used to design a tentative mechanism for LH3, as shown in Figure 13.

Figure 10 and Figure 13 show schematic representations of transition state models (TS) for tentative mechanisms for COLGAT and LH3 enzymes. In both catalytic reactions, the most pronounced TS characteristics represent a deformation of the transferred sugar that adopts half-chair/envelope conformation with shortened ring C-O bond; the anomeric carbon has *sp*^2^ hybridization and an oxo-carbenium character; the breaking glycosidic bond is longer than the standard C-O bond; and the forming glycosidic C-O linkage is considerably shorter than this distance in the reaction starting complex. How realistic the tentative mechanisms proposed for COLGALT and LH3 enzymes (Figure 10 and Figure 13) are remains to be determined.

### 6.5. Collagen Glucosidase

Protein-glucosylgalactosylhydroxylysine glucosidase (PGGHG; EC3.2.1.107) decoded by ATHL1 gene cleaves glucose from disaccharide unit α-d-glucopyranosyl-(1→2)-β-d-galactopyranose linked to hydroxylysine residues of collagen [189,190,191]. The PGGHG enzyme belongs to the glycoside hydrolase family 65 (GH65), which cleaves α-glucosidic linkages in oligosaccharides [127]. Although PGGHG is specific for disaccharide α-d-glucopyranosyl-(1→2)-β-d-galactopyranose linked to hydroxylysine, it can hydrolyze kojibiose (α-d-glucopyranosyl-(1→2)-β-d-galactopyranose) but with the five-times lower activity [189], implying a role played by collagen in the catalytic reaction. The site-directed mutagenesis of human PGGHG indicated three carboxyl residues, Asp301, Glu430, and Glu574, that are assumed to constitute the catalytic site of PGGHG [192]. The first crystal structure of a glycosidase from the GH65 family, the enzyme from Flavobacterium johnsoniae (FjGH65A), was recently solved [193]. The FjGH65A enzyme catalyzes the hydrolysis of kojibiose and other α-(1→2)-glucosides. The kinetics investigation of the catalytic mechanism revealed that FjGH65A is the α-(1→2)-glucosidase and utilizes an inverting mechanism. Although the 3D structure of PGGHG has not been solved, homology models have been generated by the developed deep-learning method AlphaFold [166,167] with a high (>89%) per-residue model confidence score and is shown in Figure 11c. 

## 7. Functional Role of Collagen Glycosylation

Collagen PTMs influence their structure, deposition, and amounts and affect cellular processes [6,14,194]. Alternations of collagen structures caused by mutation in genes encoding collagens can influence several pathological conditions. The following will discuss only the effects associated with enzymatic glycosylation. Various biological functions of collagens critically depend on the collagen interactions with numerous ligands. Collagens bind integrins and other extracellular receptors. Collagen glycan components, glucose and galactose residues, have two sides: hydrophilic and hydrophobic. The top surface containing the hydroxyl group and the ring oxygen is more polar (hydrophilic), whereas the bottom surface, which consists of hydrogens, is less polar (hydrophobic). The bottom surface is involved in interactions with hydrophobic amino acids of the protein. Hydroxyl groups usually point to the solvent and interact with surrounding molecules. Molecular modeling studies provided insights into how glycans affect the collagen structure and interactions with surrounding molecules. Molecular modeling has been performed to investigate the role of Hyl glycosylation on interactions of α2β1 and α3β1 integrins with a triple helical model of type IV collagen [195]. The results show that glycan on Hyl significantly inhibits collagen binding to integrin, most likely due to the proximity of galactose to the key interacting residues. Other molecular dynamics simulations were used to investigate how Hyl glycosylation affects the structure of type I collagen [196]. Simulations of the collagen molecular segment in an aqueous solution have shown that (a) the Hyl glycosylation does not impair triple helix structure, (b) glycosylation modulates the peptide backbone conformation and (c) hydrophilic faces of monosaccharide residues are exposed to solvent and hydrophobic surfaces point to a hydrophobic part of collagen.

Understanding the functional role played by collagen glycosylation in many cellular processes requires determining both the sites of glycosylation and the glycan structures associated with each site. This task is quite challenging, and standard structural methods, such as NMR and X-ray crystallography, are very limited for collagen. Due to recent progress, mass spectrometry (MS) has emerged as the primary tool for glycan analysis [197]. Using liquid chromatography–tandem mass spectrometry (LC-MS-MS) provides structural information with high sensitivity [198]. LC-MS-MS application offers unique semiquantitative information on collagen glycosylation that can be correlated with tissue and pathophysiological information.

### 7.1. N-Glycan Functions

The *N*-glycosylation site at the C-propeptide domain is conserved in collagens, which, together with its bulky structure, indicates that it should have essential biological functions. Although the crucial biological roles of *N*-glycans on proteins have been well described [133], it is surprising that the biological functions of the collagen *N*-glycan are still inconclusive. Docking a model *N*-glycan into the solved crystal structure of the human C-terminal propeptide domain homotrimer [199] has shown that the *N*-glycan is located on the outer face of the β-sheet and exposed to the solvent [137]. This supports the assumption that the *N*-glycan is functional. However, the C- and N-terminal pro-collagen domains are trimmed as soon as fibrillar pro-collagen is secreted into extracellular space. This suggests that the *N*-glycans have a somewhat more relevant function inside the cell than within the extracellular matrix. Surprisingly, it was found [140] that the non-glycosylated variant of C-terminal propeptide folds appropriately, similarly to the wild-type, suggesting that *N*-glycan might play a role as the signal for the clearance of trimmed C-terminal propeptides. Also, it was proposed that receptors recognize *N*-glycan for signaling to regulate collagen synthesis [200,201] or degradation [202].

Furthermore, it has been observed that the misfolding of the C-terminal pro-collagen domain is aggravated when the *N*-glycan is missing [137]. Recently, another functional proposal for *N*-glycan appeared. It was found that proper folding and secretion of procollagen in misfolding mutation requires the presence of *N*-glycosylation, and it was suggested that the critical intracellular function of *N*-glycan is to enable appropriate folding under proteostatic challenges. An absence of the *N*-glycan disturbs procollagen quality control, leading to intracellular aggregation and reduced secretion [137]. Despite the evident progress, further studies are needed to understand the functional role of collagen *N*-glycan fully. Still, available experimental data shows that *N*-glycans modulate various processes during collagen biosynthesis.

### 7.2. O-Glycan Functions

The functional role of collagen *O*-glycosylation has not been explained satisfactorily up to now. However, the progress during the last three decades indicates that *O*-glycosylation is involved in various critical structural, biological, and pathological processes. The extent of *O*-glycosylation modulates the stability of collagens against proteolytic degradation, collagen fibrillogenesis, cross-linking, secretion, assembly, and the distribution of collagens. The *O*-glycosylation also mediates interactions with non-collagenous molecules, and as the ligand for ECM components regulates specific signaling pathways, the *O*-glycosylation modulates fibril assembly and affects the morphology of fibrils [58]. It has been shown that thin fibrils were observed in collagens containing high contents of hydroxylysine. In contrast, thick fibrils have less glycosylated hydroxylysine, likely due to lesser steric interactions. The results indicate that regulation of the extent of lysine hydroxylation and hydroxylysine glycosylation may regulate the formation and morphology of the collagen fibrils. Also, fewer glycosylated collagens self-associate faster [98,203]. Since the quality and quantity of *O*-glycosylation affect the behavior of collagen in the ECM, it is evident that an appropriate folding rate is vital. It has been found that the fibrils formed from collagen with a greater extent of glycosylation had smaller diameters than fibrils formed from standard type I collagen, supporting suggestions that glycosylation alters the morphology of the formed fibrils [204]. However, another study did not observe any relationship between the level of glycosylation and the diameter of formed fibrils [205].

The *O*-glycosylation has been shown to play an essential role in forming basement membranes [172]. In that study, a loss of *O*-glycosylation in mice impaired the localization of type IV collagen and thus disrupted the formation of the basement membrane during mouse embryogenesis and led to lethality. Also, the lack of *O*-glycosylation interrupts the tetramerization and secretion of type VI collagen and the secretion of type IV collagen, implying that *O*-glycosylation is required for supramolecular assembly and collagen interactions with collagens and other proteins [206]. Together, results show that the *O*-glycosylation is fundamental for the ECM and basement membrane structure, suggesting that LH gene mutation may trigger ECM disorders and muscular diseases [207]. 

Different findings were observed on the role of *O*-glycosylation in melanoma cell invasion. In the study of melanoma cell adhesion [24], it has been observed that collagen glycosylated in the α1(IV)1263–1277 region inhibits interactions with cell-surface glycoprotein (CD44), leading to a significant decrease in their adhesion. *O*-glycosylation in the α1(IV)531-543 region also inhibits melanoma cell binding to α3β1 integrin [195]. On the other hand, the binding of the CTLD-2 lectin domain of Endo180 (recycling transmembrane glycoprotein, collagen binding receptor) to *O*-glycosylated collagen increases the endocytic efficiency of Endo-180 [208]. Since Endo-180 participates in collagen turnover during the malignant process, it enhances tumor cell invasion. Recently, an investigation of the role of collagen *O*-glycosylation in regulating carcinogenesis and metastasis by regulating cancer stem cell (CSC) phenotypes in lung cancer was carried out [209]. The results suggested that *O*-glycosylation creates a favorable microenvironment for the generation and survival of lung CSC.

Though the extracellular location is not clarified yet, some data implicate that LH3 is associated with cell growth and viability throughout cell–matrix interactions or receptor activation [210].

Experiments with the collagen-induced arthritis (CIA) model indicate that PTMs may play a vital role in autoimmune diseases by developing the T cell autoimmune response. In the CIA model, mice were immunized with recombinant type II collagens. The comparison shows that glycosylated collagen induces a higher level of arthritis than non-glycosylated collagen. The results showed that the extent of glycosylation affects the immune response to collagen [211]. 

## 8. Glycosylation Diseases

Collagens interact with many ECM components, influencing many cellular processes. Alternations in collagen structures can, therefore, affect various pathological processes, including genetic disorders, fibrosis, skin disorders, and cancer. More than 1300 collagen mutations associated with human diseases have been characterized [32]. Many excellent reviews on collagen diseases have been published where more details and further references can be found. See, for example, references [14,15,31,194,212,213,214,215,216]. Here, only collagen glycosylation-related diseases will be discussed. Recent findings that all three LH enzymes have collagen GlcGalHylT activity indicate that mutations in *PLOD1* and *PLOD2* genes may contribute to impaired hydroxylation and aberrant collagen glycosylation. Therefore, these are also mentioned here.

Undoubtedly, glycosylation is a crucial PTM of collagen and affects collagens’ structural and functional behavior. However, understanding the role of glycosylation in collagen diseases is still in its infancy and lags behind other collagen-related diseases. Several aspects of deciphering glycosylation-related diseases—the development of GTs bioassays with proper precision, accuracy, reproducibility, and operational simplicity [217]; the overlapping locations of three LHs and two GTs often complicate their characterization [113]; and the quantitative characterization of hydroxylation vs. glycosylation and their mutations [198]—have made this task challenging. However, recent advances in glycomics and MS of glycans are progressively disclosing the critical roles of glycosylation in diseases and have been reported only recently [21,58,59]. 

Various anomalies related to small blood vessels in the brain are called small-vessel diseases (SVDs) and are associated with several pathological processes [218]. Among clinical signs are ischemic strokes, a more significant proportion of intracerebral hemorrhages and causes of death, and long-term severe disability. Mutations in *Col4a1* [219,220] and *Col4a2* [221] genes are associated with SVDs. These mutations lead to the impaired secretion and intracellular accumulation of collagens COL4A1 or COL4A2 [220,221], affecting the quality of the basement membrane. Recently, in two unrelated patients with SVDs, biallelic variants in the *COLGALT1* gene encoding ColGalT1 have been discovered [222]. Both patients showed clinical symptoms similar to those in *Col4A1/Col4A2* disease [219]. The study showed that impaired GalHylT activity decreases *COL4A1* production. As a result, the secretion of type IV collagen into extracellular space was disrupted. This might contribute to basement membrane fragility, causing cerebral small vessel aberration similar to the pathological characteristics of *Col4A1/Col4A2* mutants. 

Connective tissue disorder (CTD) due to LH3 deficiency (bone fragility–contractures-arterial rupture–deafness syndrome) is a rare genetic disease caused by a lack of LH3 activity. The disease involves multiple tissue and organ involvement, including skeletal abnormalities; ocular involvement; and hair, nail, and skin anomalies. Patients also present intrauterine growth retardation, facial dimorphism, and joint flexion contractures. Growth and developmental delay, bilateral sensorineural deafness, friable diaphragm, and later-onset spontaneous vascular ruptures are additional reported features [223]. In two patients with CTD clinical symptoms, a significantly reduced GlcGalHylT activity was observed in serum [224,225,226]. Analyses revealed two heritable *PLOD3* mutations, one from the father and the second from the mother. The locations of mutations were found in regions with glycosyltransferase and lysyl hydroxylase activities. The observed data show mainly type II collagen disorders, suggesting the molecular basis of the CTD syndrome. The consequences of small changes in nucleotides of the *PLOD3* gene are dramatic, with all activities of LH3 considerably reduced [224].

Mutations in the genes encoding LH cause several severe or lethal diseases. The Ehlers–Danlos syndrome (EDS) and Nevo syndrome [227] are a group of heritable connective tissue disorders characterized by skin hyperextensibility, articular hypermobility, and tissue fragility. The significant characteristics of kyphoscoliotic-type EDS are severe muscle hypotonia, generalized joint laxity, scoliosis, and scleral fragility and the rupture of the ocular globe [228,229]. At least 20 different mutations have been identified in *PLOD1* [230] and *PLOD3* [224], causing a deficiency in lysyl hydroxylase. 

Bruck syndrome is an autosomal recessive syndrome consisting of bone fragility and congenital joint contractures. According to the genotype, it has been classified into types 1 and 2 involving mutations of the *FKBP10* and *PLOD2* collagen genes, respectively [231,232,233]. For example, data from two siblings, a six-year-old male and a five-year-old female, with Bruck syndrome type 2 of non-affected parents, were recently published [234]. The male has experienced more than 45 fractures, developed severe scoliosis, and has debilitating flexion contractures. The female has minimal flexion contractures, a history of 15 fractures, and severe scoliosis. *PLOD2* mutations result in a dramatic underhydroxylation of collagen telopeptide Lys, causing a decrease in the pyridinoline cross-linking of type I collagen.

Epidermolysis bullosa (EB) simplex [207,224,235] is a group of rare heritable diseases that cause the skin to be fragile and to blister easily. Tears, sores, and blisters happen when something rubs or bumps the skin. Mutations of around ten genes among *PLOD3* [207,224,226,236] inherited from parents cause most forms of epidermolysis bullosa. In patients, the pathological mutation in *PLOD3* led to a decreased expression of LH3 [236] and type VII collagen in the skin, resulting in reduced glucosylation [226]. It was suggested that the decrease in GlcGalHylT activity correlates with the severity of phenotype and the resulting abnormalities in the organization of the extracellular matrix [207].

Stickler syndrome is a group of hereditary conditions characterized by a distinctive facial appearance, eye abnormalities, hearing loss, and joint problems. Stickler syndrome is a genetic condition affecting connective tissues in the face and joints, leading to problems with vision, hearing, and movement problems. This hereditary condition is usually diagnosed in babies and children, and early treatment leads to a positive outcome [237]. Stickler syndrome is caused by mutations in the *COL2A1* and *COL1A1* genes that are associated with the formation of types II, IX, and XI collagens and affect their structure and properties [238,239], and also by mutation of the *PLOD3* gene [224,225]. Affected persons show a decreased glucosyltransferase activity. Although clinical symptoms best describe Stickler syndrome, they also exhibit variable features of EDS and EB [225].

In addition to heritable connective tissue diseases, impairments of glycosylation are associated with various common diseases, and some will be briefly mentioned below. Covalent intermolecular cross-links are the final step in collagen biosynthesis and affect many biomechanical functions of collagen [58]. This process starts after the secretion of collagen into extracellular space by the action of LOX and is followed by spontaneous interactions between neighboring chains (see paragraph on LOX). This process is critical for the properties of fibrils, influences fibrinogenesis and matrix mineralization, and is affected by the glycosylation of collagen [98,99].

An increase in the glycosylation of the collagen triple helix has been shown in degenerative joint disease (osteoarthritis cartilage) [240]. A comparison of lysyl hydroxylation and pyridinoline cross-linking of normal and degenerative knee showed that degenerative cartilage contained significantly more hydroxylysine residues per collagen molecule than healthy cartilage from the same donor. 

Inflammation or irritation of a tendon (tendinitis) occurs in the elbow, knee, shoulder, and hip. In a normal tendon, type I collagen accounts for almost 95% of the total collagen [241]. In tendinitis, the previously functional collagen is replaced by aberrant collagen with a lower ratio of pentosidine and higher ratios of hydroxylated lysine residues in collagen cross-links [242,243]. More hydroxylated lysine residues were also present in Achilles tendinosis [244]. 

Losing bone mineral density weakens bones and can lead to osteoporosis (osteopenia). It was found that the degree of the lysyl hydroxylation of both α-chains of type I collagen showed a significant inverse correlation with the trabecular bone volume. Also, the extent of the glycosylation of type I collagen correlated with both the level of lysylhydroxylation and the degree of osteopenia [205].

The extent of glycosylation has been found to influence metastatic cancer. Collagen cross-link accumulation increases stromal stiffness and stimulates tumor cells’ invasive properties. For example, tumor stroma contains higher levels of hydroxylysine aldehyde-derived collagen cross-links and lower levels of lysine aldehyde-derived cross-links compared to normal lung tissues due to the higher expression of LH2, which indicates that LH2 enhances the collagen metastatic properties of tumor cells and functions by controlling the relative abundance of hydroxylysine aldehyde-derived collagen cross-links in the tumor stroma [245]. The role of LH2 in metastasis is supported by pharmacologic inhibition of the *PLOD2* enzymatic activity that suppresses metastases. These data indicate that *PLOD2*-dependent collagen modifications influence metastasis [246]. Also, the higher expression of the *PLOD1* and *PLOD2* genes was found in hypoxic breast cancer cells and is associated with an increased risk of mortality. It was suggested that *PLOD2* is critical for fibrillar collagen formation by breast cancer cells, increases tumor stiffness, and is required for metastasis to lymph nodes and lungs [247].

## 9. Concluding Remarks

Post-translational modifications are the essential characteristics of collagen, including proline and lysine hydroxylation, *N*-glycosylation, the *O*-glycosylation of Hyl, the oxidative deamination of Lys and Hyl residues in the telopeptide domains, and following intra and intermolecular covalent cross-linking. The hydroxylation of proline in positions 3 and 4 is crucial for the stability of the triple-helical structure, whereas the hydroxylation of lysine residues is a prerequisite for subsequent glycosylation. The glycosylation of collagen takes place in the ER before forming a triple-helical assembly. It is simple but unique since the attached *O*-glycan comprises either monosaccharide galactose or disaccharide α-d-glucopyranosyl-(1→2)-β-d-galactopyranose. Collagen *O*-glycosylation commences by transferring the galactose residue from the donor UDP-Gal to the 5-hydroxyl group of Hyl by the β-glycosidic linkage. Two inverting galactosyltransferases, COLGALT1 and COLGALT2, catalyze this transfer. The second step is the transfer of the glucose residue from the donor UDP-Glc to the C2 hydroxyl of glucose by the α-glycosidic linkage through the catalytic action of a multifunctional enzyme lysylhydroxylase LH3 utilizing the retaining mechanism. The extent of glycosylation, glycosites, and the heterogeneity of glycan distribution (mono versus disaccharide) depend on the collagen types, tissue, cell type, maturation stage, and pathological condition and seem to modulate collagen structural organization and function in the ECM.

Although the functional role of collagen glycosylation and its involvement in diseases is not fully understood, it is clear that collagen *O*-glycosylation is fundamental for its correct assembly and ECM deposition. Many studies showed that collagen aberrant *O*-glycosylation is associated with connective tissue disorders, cancer development, and metastasis. Collagens also play a crucial role in the properties of basement membranes, where their impaired deposition can cause pathological conditions. There are indications that *O*-glycosylations modulate the physiological cross-linking catalyzed by the enzyme lysyl oxidase. Primarily, four carbohydrate processing enzymes, namely COLGALT1, COLGALT2, LH3, and PGGHG, are involved together with lysyl hydroxylases in the biosynthesis of *O*-glycan, where a delicate balance between their actions defines the *O*-glycosylation pattern. Recent progress in the understanding of the catalytic mechanism of glycosyltransferases and glycosidases, together with progress in collagen glycosylation analysis by mass spectrometry and biochemical characterization, offer tools to decipher the details of collagen *O*-glycosylation and its micro- and macro-heterogeneity. Such a multidisciplinary approach can contribute to fully unraveling glycosylation’s role in collagen’s structure and function. This knowledge will provide a rational guide to treating *O*-glycosylation-related diseases and to the development of new therapeutic agents for cancer, fibrosis, and regenerative medicine.
